# Liquid-based cytology for the detection of cervical intraepithelial lesions in Jimma town, Ethiopia

**DOI:** 10.1186/s12885-020-07201-9

**Published:** 2020-07-29

**Authors:** Getnet Tesfaw, Yesuf Ahmed, Lealem Gedefaw, Lamessa Dube, Samson Godu, Kirubel Eshetu, Mesfin Nigussie, Haftamu Hailekiros, Moses Joloba, Gelila Goba, Alemseged Abdissa

**Affiliations:** 1 Jimma University, School of Medical Laboratory Sciences, PO Box =378, Jimma, Ethiopia; 2 Jimma University, Department of Obstetrics & Gynecology, Jimma, Ethiopia; 3 Jimma University, Department of Epidemiology, Jimma, Ethiopia; 4 International Clinical Laboratories, Addis Ababa, Ethiopia; 5 Mekelle University, Department of Medical Microbiology and Immunology, Mekelle, Ethiopia; 6 Department of Microbiology, Makerere University, Kampala, Uganda; 7 Department of Obstetrics and Gynecology, University of Illinois at Chicago, Chicago, USA

**Keywords:** Liquid-based cytology, LBC, VIA, Cervical squamous intra-epithelial lesions, Ethiopia

## Abstract

**Background:**

Cervical cancer is the second leading type of female cancer in Ethiopia. Screening for cervical cancer is primarily conducted using visual inspection with 5% acetic acid (VIA). Liquid-based cytology (LBC) is not yet widely used in Ethiopia.

**Method:**

Women aged 21–65 years were tested using LBC and VIA to detect cervical dysplasia. Logistic regression analysis was conducted to identify associated factors. Cohen’s Kappa test was conducted to test agreement between LBC and VIA.

**Results:**

Forty-two percent (*n* = 188) of 448 participants were 31 to 40 years of age and only two participants were above 60. Of the 448 participants, 419 (93.5%) were tested with LBC, 294 (65.6%) VIA and 272 (60.7%) with both LBC and VIA. Among women screened using LBC, 305 (72.8%) were negative for intraepithelial lesion or malignancy (NILM), 97 (23.2%) had low-grade squamous intraepithelial lesion (LSIL) and 17 (4.1%) had high-grade squamous intraepithelial lesion (HSIL). Presence of cervical lesions was generally lower in younger and older women. Majority, 39 (40%) of women with LSIL and 10 (59%) with HSIL were 41–50 years of age. Women aged 51–60 were more likely to have abnormal intraepithelial lesions compared to women aged 21–30 (AOR = 20.9, 95% CI = [7.2–60.9], *p* = 0.00). Out of 47 (10.8%) HIV-positive women, 14 (32.56%) had intraepithelial lesions of which 10 (23.3%) and 4 (9.3%) had LSIL and HSIL, respectively. Among women screened with VIA, 18 (6.1%) were positive; among the 272 (60.7%) women screened using both LBC and VIA, 6 (2.2%) were positive on both LBC and VIA tests. The level of agreement between the two tests was weak at a statistically significant level (kappa value = 0.155, *p* = 0.006).

**Conclusion:**

LBC demonstrated high rates of cervical squamous intra-epithelial lesions in our study. VIA was a less reliable predictor of cervical squamous intra-epithelial lesions than LBC. Evaluating diagnostic accuracy of both LBC and VIA against a histological endpoint should be completed before adopting either or both screening modalities.

## Background

Human papilloma virus (HPV) is the most common sexually transmitted infection (STI) in the world [1]. HPV causes a variety of malignancies, with cervical cancer being the most important and prevalent [2]. Cervical cancer is a leading public health challenge globally, with 569, 847 women were diagnosed and 311,365 women dying from the disease in 2018 [3]. Majority (85%) of deaths occurred in low- and middle-income countries [4]. In Africa, 119, 284 new cases of cervical cancer were diagnosed and 81, 687 women died in 2018. The highest rate of cervical cancer was found in eastern and western region of Africa [3].

In Ethiopia, 5.8% of national mortality is attributable to cancer and incidence is increasing because of the aging population. Cervical cancer is the second leading cause of female cancer in women aged 15 to 44 in Ethiopia [5]. According to the Global Cancer Observatory, 6294 new cases were diagnosed and 4884 women died from the disease in 2018 [6]. According to the Ethiopian Ministry of Health, approximately 80% of reported cases of cancer are diagnosed at advanced stages [5].

In 2016, Ethiopia introduced a national cancer control plan that includes using visual inspection with acetic acid (VIA) and corresponding treatment of women testing positive. VIA continues to be the only cervical cancer screening modality in the country [5]. There is no organized cytology-based cervical lesion screening program in Ethiopia. As per the researchers’ knowledge, no cervical cancer screening has ever been conducted in Ethiopia that combines LBC and VIA. This study documents the burden of cervical lesions and predictors of abnormal cervical cytology as well as comparing LBC and VIA screening modalities.

## Methods

### Study design and setting

A cross sectional study was conducted in Jimma Town from February 2017 to May 2018. Jimma is located 350 km southwest of Addis Ababa. A total of 448 non-pregnant women ages 21–65 who visited Jimma University Hospital as well as Marie Stopes International and Family Guidance Association of Ethiopia (FGAE) Clinics for VIA screening as part of the national cervical cancer screening program were enrolled consecutively. Women with complete hysterectomy, gross tumor on the cervix, prior surgeries involving the cervix, those who were menstruating, and those with no history of sexual activity were excluded. Written informed consent was obtained and the procedure of the test was explained to women.

### Demographic and risk factors

Demographic information and risk factors for cervical cancer were collected using questionnaires prepared in *Afan Oromo* and *Amharic* languages*.* The collected data included occupation, educational status, age, parity, marital status, history of contraceptive use, age at first sexual intercourse, smoking habit, number of lifetime sexual partners, family history of cervical cancer, STIs and alcohol consumption.

### Liquid-based cytology (LBC)

An automated liquid-based cytology, SurePath™ liquid-based Pap test (BD, USA), was employed for cytological sample preparation. After removing obscuring mucus from the cervix with a cotton swab, endocervical and ectocervical cells were collected with cytobrush. This cytobrush was immediately rinsed in a vial containing SurePath Preservative Fluid. Samples were transported at room temperature for analysis at the International Clinical Laboratories (ICL) in Addis Ababa using BD PrepMate™ and PrepStain™ Slide Processor. Vials containing samples were labeled and placed into the BD PrepMate™ Slide Processor in which a liquid-based filtration process removed mucus and debris, preserving cell morphology, and making a smear of even distribution. All slides were stained with the BD SurePath Kit Cytology Stain and examined by two pathologists [7] who were enrolled in the College of American Pathologists (CAP) proficiency program and received stained LBC slides every three months as well as participating in the external quality assurance scheme. LBC test results were recorded based on the Bethesda gynecologic cytology guideline [8].

### Visual inspection with acetic acid (VIA)

Women visited health facilities in Jimma Town involved in national cervical cancer screening program were enrolled for VIA. Women with invisible transformation zone were excluded from the study. After obtaining informed consent, a sterile plastic spatula was inserted into the vagina to visualize the cervix. Then, 5% acetic acid was applied to the cervix for one minute. Positive test was defined as a “sharp, distinct, well-defined, dense (opaque, dull or oyster white) aceto-white area with or without raised margins” according to the standard guideline [9, 10]. VIA examination was done by experienced clinical nurses who participated in the national cervical cancer screening program using VIA.

### Data analysis

Data was checked for completeness, coded and entered into EpiData v3.1 and exported to Stata^14^ for analysis. Descriptive statistics, frequency and proportion were used to describe demographic variables. Sub-group analysis was conducted for HIV patients. Logistic regression analysis was used to identify risk factors for abnormal cervical cytology on the LBC test. Cohen’s Kappa test was used to assess agreement between LBC and VIA test (*p*-value < 0.05 was considered statistically significant at 95% confidence).

## Results

### Characteristics of study participants

Mean age of participants was 38 (SD = ±9) and ranged from 21 to 65. Forty-two percent (*n* = 188) of women fell between 31 and 40 years of age. Only two participants were above 60 years of age. Three hundred thirty-three (74.3%) women were married. One hundred ninety-four (44.5%) had sexual debut between the ages of 10 and 17. Thirty-nine (8.8%) were primigravida and 339 (74.1%) multigravida and 163 (38.3%) had two or more lifetime partners. Seventy-eight (17.7%) had history of STIs and 47 (10.8%) were HIV patients on ART follow-up at Jimma University’s TB-HIV clinic [Table 1].

**Table 1:** Abnormal intraepithelial lesions by LBC and demographic characteristic, Jimma, 2018

Characteristics		N (%)	Liquid-based cytology (LBC) Result		
			NILM	LSIL	HSIL
			n(%)	n(%)	n(%)
Age	21–30	105 (25.06)	97 (92.38)	8 (7.62)	0 (0.00)
	31–40	179 (42.72)	144 (80.45)	30 (16.76)	5 (2.79)
	41–50	102 (24.34)	53 (51.96)	39 (38.24)	10 (9.80)
	51–60	31 (7.40)	11 (35.48)	18 (58.06)	2 (6.45)
	> = 61	2 (0.48)	0 (0.00)	2 (100.00)	0 (0.00)
Occupation	Government worker	159 (38.8)	100 (62.89)	46 (28.93)	13 (8.18)
	Merchant	30 (7.3)	25 (83.33)	4 (13.33)	1 (3.33)
	Student	11 (2.7)	10 (90.91)	1 (9.09)	0 (0.0)
	Housewife	164 (40.1)	127 (77.44)	34 (20.73)	3 (1.83)
	Other	45 (11)	36 (80.00	9 (20.00)	0 (0.00)
Educational Status	Illiterate	84 (20.14)	57 (67.86)	26 (30.95)	1 (1.19)
	Primary	110 (26.38)	91 (82.73)	15 (13.64)	4 (3.64)
	Secondary	91 (21.82)	66 (72.53)	23 (25.27)	2 (2.20)
	University	132 (31.65)	90 (68.18)	32 (24.24)	10 (7.58)
Marital Status	Married	313 (74.88)	240 (76.68)	62 (19.81)	11 (3.51)
	Single	23 (5.50)	20 (86.96)	3 (13.04)	0 (0.00)
	Divorced	28 (6.70)	22 (78.57)	5 (17.86)	1 (3.57)
	Other	54 (12.92	23 (42.59)	26 (48.15)	5 (9.26)
Parity	Nulligravida	34 (8.17)	29 (85.29)	3 (8.82)	2 (5.88)
	Primigravida	70 (16.83)	55 (78.57)	13 (18.57)	2 (2.86)
	Multigravida	312 (75.00)	219 (70.19)	80 (25.64)	13 (4.17)
Menstrual Bleeding Pattern	Irregular	151 (37.1)	131 (86.75)	18 (11.92)	2 (1.32)
	Regular	120 (29.48)	111 (92.50)	9 (7.50)	0 (0.00)
	Menopause	136 (33.42)	53 (38.97)	68 (50.00)	15 (11.03)
Postcoital bleeding	No	377 (93.32)	268 (71.09)	92 (24.40)	17 (4.51)
	Yes	27 (6.68)	24 (88.89)	3 (11.11)	0 (0.00)
Age of first sexual intercourse	10–17	183 (44.74)	132 (72.13)	45 (24.59)	6 (3.28)
	> = 18	226 (55.26)	166 (73.45)	49 (21.68)	11 (4.87)
Use of contraceptive	No	305 (73.14)	203 (66.56)	87 (28.52)	15 (4.92)
	Yes	112 (26.86)	100 (89.29)	10 (8.93)	2 (1.79)
Current sexual partner	No	138 (33.17)	87 (63.04)	43 (31.16)	8 (5.80)
	Yes	278 (66.83)	216 (77.700	53 (19.06)	9 (3.24)
Condom use during sexual intercourse	No	392 (94.23)	282 (71.94)	95 (24.23)	15 (3.83)
	Yes	24 (5.77)	21 (87.50)	1 (4.17)	2 (8.33)
Alcohol use	No	384 (93.20)	276 (71.88)	91 (23.70)	17 (4.43)
	Yes	28 (6.80)	24 (85.71)	4 (14.29)	0 (0)
Smoking	No	417 (99.52)	303 (72.66)	97 (23.26)	17 (4.08)
	Yes	2 (0.48)	2 (100)	0 (0.00)	0 (0.00)
Chronic corticosteroid use	No	396 (96.82)	292 (73.74)	87 (21.97)	17 (4.29
	Yes	13 (3.18)	6 (46.15)	7 (53.85)	0 (0.00)
Number of lifetime sexual partners	1	247 (61.75)	174 (70.45)	63 (25.51)	10 (4.05)
	> = 2	153 (38.25)	116 (75.82)	30 (19.610	7 (4.58)
History of sexually transmitted diseases	No	339 (82.48)	246 (72.57)	80 (23.60)	13 (3.83)
	Yes	72 (17.52)	53 (73.61)	16 (22.22)	3 (4.17)
HIV status	Non-reactive	135 (33.09)	104 (77.04)	26 (19.26)	5 (3.70)
	Reactive	43 (10.54)	29 (67.44)	10 (23.26)	4 (9.30)
	Unknown	230 (56.37)	163 (70.87)	59 (25.65)	8 (3.48)
Family history of cancer	No	350 (94.85)	254 (72.57)	80 (22.86)	16 (4.57)
	Yes	19 (5.15)	14 (73.68)	5 (26.32)	0 (0.00)
Pelvic examination	Abnormal	7 (1.78)	5 (71.43)	1 (14.29)	1 (14.29)
	Normal	386 (98.22)	284 (73.58)	86 (22.28)	16 (4.15)
SCJ visible	No	120 (33.06)	60 (50.00)	47 (39.17)	13 (10.83)
	Yes	243 (66.94)	207 (85.19)	32 (13.17)	4 (1.65)

### Intraepithelial lesions screening by LBC and VIA

Of 448 participating women, 28 (6.3%) were missing LBC results and 1 (0.22%) had an inadequate sample. The remaining 419 (93.5%) women had LBC results, 294 (65.6%) had VIA results and 272 (60.7%) had both LBC and VIA results.

Among women screened using LBC, 305 (72.8%) tested negative for intraepithelial lesion or malignancy (NILM), 97 (23.2%) had LSIL and 17 (4.1%) had HSIL. No ASC-US, ASC-H or squamous carcinoma was present. Cervical lesions consisting of either LSIL or HSIL were present in 114 (27.2%) women. Presence of cervical lesions was generally lower in younger and older women compared to middle-aged women. Among women with cervical lesions, 8 (7%) were below the age of 31 and 2 (1.8%) were over 60. Of the remaining women, 70 (30.7%) were 31–40, 49 (43%) were 41–50, and 20 (17.5%) were 51–60 years of age. Thirty-nine (40%) women with LSIL and 10 (59%) with HSIL were between 41 and 50 years of age. Among the 419 women tested using LBC, 120 (33.1%) had invisible SCJ during examination. Of these, 60 (50%) had either LSIL or HSIL [Table 1].

Two hundred seventy-two (60.7%) women were screened using both LBC and VIA. Among women screened using VIA, 18 (6.1%) tested positive. Eleven (4.7%) of these were among the 236 (86.8%) cases recorded as NILM by using LBC. Of the 36 (12.1%) women who had either LSIL or HSIL on the LBC test, 30 (83.3%) tested negative on the VIA test. No women with HSIL tested positive using VIA. There was no agreement between the two screening tests using Cohen’s Kappa test (kappa value = 0.155, *p* = 0.006) [Table 2].

**Table 2:** Cervical lesion abnormality among women screened by both LBC and VIA, Jimma, 2018

LBC Result	VIA Test Result		Total N(%)	Kappa value	P-value
	Negative n(%)	Positive n(%)		0.155	0.006
NILM	225 (95.34)	11 (4.66)	236 (100)		
LSIL or HSIL	30 (83.33)	6 (16.67)	36 (100)		
Total	255 (93.75)	17 (6.25)	272 (100)		

### Characteristics of HIV patients

A total of 47 HIV patients on ART who visited Jimma University Cervical Cancer Clinic were screened for cervical lesions. Of these, 21 (45.7%) were married, 15 (31.9%) were primigravida and 28 (59.6%) multigravida and 23 (48.9%) had sexual debut between the ages of 11 and 17. Thirty-two (68.1%) had multiple sexual partners and 15 (32%) had history of STIs.

Twenty-eight (59.6%) and 43 (91.5%) HIV-positive women were tested with VIA and LBC, respectively. Only two HIV-patients were positive on the VIA test (7.1%) whereas 14 (32.6%) had either LSIL or HSIL on the LBC test. Of this latter group, 10 (23.3%) and 4 (9.3%) had LSIL and HSIL, respectively. Among HIV-positive women between the ages of 41–50, 7 (77.78%) had intraepithelial lesions. Half of HIV-patients with interepithelial lesions were between the ages of 41–50.

### Predictors of abnormal cytology by LBC

Bivariate logistic regression analysis revealed that parity, age and condom use during sexual intercourse were significant for inclusion in the multivariate regression analysis at p = < 0.25. Multivariate regression revealed that age was an independent predictor of LSIL and HSIL. Odds of being positive for cervical squamous-extraepithelial lesions were higher in women older than 31 years of age. Women 51–60 years of age were more likely to have abnormal intraepithelial lesion compared to women aged 21–30 (AOR = 20.9, 95%CI = [7.2–60.9], *p* = 0.00) [Table 3].

**Table 3:** Predictors of abnormal cervical cytology using LBC, Jimma, 2018

Characteristics		N(%)	LBC Result		COR(95%:CI)	***P-Value***	AOR(95%: CI)	***P-Value***
			NILM	LSIL or HSIL				
			n (%)	n(%)				
Parity	Nulligravida	34 (8.17)	29 (85.29)	5 (14.71)	1(ref.)		1	
	primigravida	70 (16.83)	55 (78.57)	15 (21.43)	1.6 (0.52–4.8)	0.42	1.4 (0.43–4.9)	0.56
	Multigravida	312 (75)	219 (70.19)	93 (29.81)	2.5 (0.9–6.6)	0.07	1.1 (0.38–3.3)	0.85
Age	21–30	105 (25.06)	97 (92.38)	8 (7.62)	1(ref.)		1	
	31–40	179 (42.72)	144 (80.45)	35 (19.55)	2.9 (1.3–6.6)	0.00	2.9 (1.3–6.8)	0.00
	41–50	102 (24.34)	53 (51.96)	49 (48.04)	11.2 (4.9–25.4)	0.00	11.4 (4.8–26.9)	0.00
	51–60	31 (7.40)	11 (35.48)	20 (64.52)	22.0 (7.8–61.5)	0.00	20.9 (7.2–60.9)	0.00
	> = 61	2 (0.48)	0 (0.00)	2 (100.00)	1			
Condom Use	No	392 (94.23)	282 (71.94)	110 (28.06)	2.7 (0.79–9.3)	0.12	1	
	Yes	24 (5.77)	21 (87.50)	3 (12.50)	1(ref)		1.9 (0.52–6.9)	0.33

## Discussion

In Ethiopia, 29 million women are over 14 years of age and many of these women are at risk of developing cervical cancer [5]. In 2018, 6294 women were diagnosed as new cervical cancer cases and 4884 women died from the disease [6].

Even though cervical cancer burden is high in Ethiopia, the national cancer screening program is based solely on VIA, which has high variability due to examiners’ judgment [11]. Our study is the first to show results of cervical cancer screening in Ethiopia using LBC. In our study, abnormal squamous intraepithelial lesion was present in 114 (27%) women, which is higher than the 17% of women that were positive in a study in China [12]. Prevalence of LSIL and HSIL were 23.2% and 4.1%, respectively, in our study, much higher than the 1.9% and 0.6% prevalence, respectively, observed in Sao Paulo [13]. A study in India reported a lower rate of LSIL (7.5%) than our study, but higher HSIL (10.5%) [14]. Significantly, lower prevalence of LSIL and HSIL (2%) and (2.4%) were observed in the Netherlands and Germany, respectively, [15, 16]. Low rates in developed countries may be due to the availability of the HPV vaccines [17] and the presence of organized cervical cancer screening [18], which is new to Ethiopia.

In our study, a higher proportion of women aged 41–50 tested positive on the LBC screening test. In contrast, we observed lower prevalence of cervical lesions in younger and older women. Visibility of SCJ is the prerequisite for VIA examination and women with invisible SCJ are exempt for VIA examination [19]. In our study, women with invisible SCJ, underwent LBC testing and 60 (50%) had either HSIL or LSIL on the LBC test.

Logistic regression showed women aged 51–60 had higher odds of having cervical squamous intraepithelial lesions compared to younger women. LBC screening was better at detecting HSIL and cervical lesions in older women, which is not true for VIA screening [20].

HIV infection is a risk factor for persistent HPV-infection, a necessary condition for the development of squamous interepithelial lesions. HIV-positive women are disproportionately affected by cervical lesions [21]. In our study, 14 (32. 6%) HIV-patients had cervical squamous intraepithelial lesions, which is higher than prevalence in the total study population (27%). While the rate of LSIL (23.3%) among HIV-positive women was similar to the full study cohort, the prevalence of HSIL (9.3%) among HIV-positive women was nearly double the study cohort. A study in South Africa recorded higher prevalence of LSIL (32.5%) and HSIL (23.3%) than our study [22] whereas a study in Nigeria showed LSIL and HSIL rates to be 14.3% and 4.3%, respectively, among HIV-positive women [23].

VIA detected 18 (6.1%) cases of cervical lesions, which is similar to the 4.7% reported in Butajira, Ethiopia [24], but lower than the 12.9% reported in another study in Jimma Town [25] as well as studies in Rwanda and China, where 14.7% [26] and 11.4% [12] of women, respectively, had cervical lesions. Among women who were tested using both LBC and VIA in our study, a high proportion (83.3%) that tested positive using the LBC test tested negative on the VIA test. This finding is similar to a study in China that showed VIA missed the majority of CIN2+ in older women and was less sensitive than LBC [12]. As our study showed, there was no agreement between LBC and VIA screening tests and variability in the tests was statistically significant (kappa =0.155, *P* = 0.006).

Organized cytology-based screening is the most efficient screening method for the detection of cervical lesions and has resulted in significant reduction in cervical cancer in developed countries [27]. Financial constraints and technical challenges hinder implementing cytology-based screening in low- and middle-income countries like Ethiopia.

## Conclusion

Given that VIA screening missed most cervical lesions detected by LBC in our study and that a high number of cervical epithelial lesions were detected by LBC, a larger study should be undertaken to determine the diagnostic accuracy of both LBC and VIA against a histological endpoint before adopting either or a combination of the two as screening modalities.

## Data Availability

The datasets used and/or analyzed during the current study are available from the corresponding author on reasonable request.

## Abbreviations

VIA: Visual inspection with 5% acetic acid
LBC: Liquid-based cytology
HPV: Human papilloma virus
FGAE: Family Guidance Association of Ethiopia
NILM: Negative for intraepithelial lesion or malignancy
LSIL: Low grade squamous intraepithelial lesion
HSIL: High grade squamous intraepithelial lesion
SCJ: Squamous columnar junction
ICL: International Clinical Laboratories
STIs: Sexually transmitted infections
